# Neuroprotective effects of daidzein against ifosfamide-induced neurotoxicity in male rats: role of selected inflammatory and apoptotic markers

**DOI:** 10.25122/jml-2023-0082

**Published:** 2023-11

**Authors:** Hiba Zaki Hammoodi, Nada Naji Al-Shawi

**Affiliations:** 1 Department of Pharmacology and Toxicology, College of Pharmacy, University of Baghdad, Baghdad, Iraq

**Keywords:** Ifosfamide (IFO), daidzein (DZN), neurotoxicity, DZN: Daidzein, IFO: Ifosfamide, IL-6: Interleukin-6, IL-10: Interleukin-10, iNOS: Inducible nitric oxide synthase, TNF-α: Tumor necrosis factor-alpha

## Abstract

Ifosfamide (IFO), an alkylating chemotherapy agent, is known for its association with neurotoxicity and encephalopathy. This trial was designed to evaluate the protective action of daidzein (DZN) against IFO-induced neurotoxicity in male rats by determining the difference in certain inflammatory and apoptotic markers in the brain tissue of rats. Twenty-eight Wistar rats, weighing 120-150 g, were divided into four groups of seven rats: Group 1 (Control) received no treatment; Group 2 was orally administered DZN (100 mg/kg/day) for seven days; Group 3 received a single intraperitoneal (IP) dose of IFO (500 mg/kg); Group 4 received oral DZN (100 mg/kg/day) for one week prior to a single IP dose of IFO on the seventh day. Twenty-four hours post-treatment, serum and brain tissue samples were collected for analysis. The results indicated a significant increase in serum inflammatory markers (TNF-alpha, IL-6, and iNOS) and the anti-inflammatory marker (IL-10), along with elevated caspase-3 enzyme activity in the brain tissue of the IFO-treated group compared to the control group. Conversely, pre-treatment with DZN significantly reduced serum inflammatory markers and caspase-3 levels in tissue. The findings suggest that daidzein has anti-inflammatory and anti-apoptotic properties, potentially offering protection against IFO-induced neurotoxicity in rats.

## INTRODUCTION

Neurotoxicity refers to any harmful effect on the chemistry, structure, and function of the central nervous system (CNS) or peripheral nervous system (PNS) resulting from exposure to toxic substances. These naturally occurring or artificially synthesized substances can disrupt or damage the nerves responsible for processing and transmitting information in the brain and other parts of the nervous system [1].

Ifosfamide (IFO), an alkylating agent used in chemotherapy, is used for managing a variety of neoplasms such as sarcoma, lymphoma, and germ cell tumors [2]. However, this chemotherapy drug can cause several unwanted effects, including alopecia, arrhythmias, interstitial pneumonitis, bone marrow suppression/myelosuppression, hemorrhagic cystitis, fatigue, disorientation, blurred vision, seizures, and auditory or visual paranoid hallucinations [3-5]. According to statistics, IFO-induced neurotoxicity is expected in 3-19% of children and 10-30% of adults [6-8]. The first signs of neurotoxicity are often observed between 12 and 146 hours after IFO administration, and they typically diminish on their own within three days after IFO is discontinued [7]. Research has suggested that a metabolite of IFO, chloroacetaldehyde, is involved in the pathogenesis of neurotoxicity [9]. The neurotoxic effects caused by these metabolites are due to a reduction in the level of glutathione in the CNS and the cessation of oxidative phosphorylation in the mitochondria, resulting in disturbances in fatty acid metabolism [8]. Reducing the harmful effects of chemotherapy on healthy cells is critical [10]. Therefore, natural substances that can effectively mitigate IFO-induced neurotoxicity are needed. Daidzein (DZN), a polyphenolic isoflavone compound obtained from red clover (*Trifolium pratense*), alfalfa (*Medicago sativa*), soy, and various legumes from the *Leguminosae* family, has anti-inflammatory, cardioprotective, and antioxidant properties and is protective against breast, prostate, and colorectal cancers [11-14].

This study aimed to investigate the anti-inflammatory and anti-apoptotic effects of DZN against IFO-induced neurotoxicity, focusing on various biochemical parameters.

## MATERIAL AND METHODS

### Drugs, chemicals, and kits

IFO powder was sourced from Picasso, China. Daidzein (DZN) in pure powder form was procured from Macklin Company. Phosphate-buffered saline (PBS) was obtained from Santa Cruz Biotechnology, USA. Enzyme-linked immunosorbent assay (ELISA) kits specific to rats, including tumor necrosis factor-alpha (TNF-α), interleukin-6 (IL-6), interleukin-10 (IL-10), inducible nitric oxide synthase (iNOS), and caspase-3 (Casp-3), were purchased from Nanjing Pars Biochem CO. Diethyl ether, used as an anesthetic, was acquired from Erochem Limited.

### Animals

Twenty-eight adult male Wistar rats weighing between 120-150g were procured from the animal house at the College of Pharmacy, University of Baghdad. They were housed under standard conditions (controlled temperature, humidity, and light/dark cycles) at the Animal Experimental and Scientific House of the College of Pharmacy, Baghdad University. Rats were fed commercial pellets and tap water ad libitum during the trial time and were accommodated for one week before the experiment.

### Experimental design

This trial was approved by the Scientific and Ethical Committees of the College of Pharmacy/University of Baghdad. The rats were divided into four groups, each comprising seven animals:

**Control group:** Rats received an oral administration of 1% tween 20 dissolved in distilled water (DW) daily for seven days through oral gavage.

**DZN group:** Rats were orally administered DZN (100 mg/kg/day) [15] suspended in DW using 1% tween 20 as a surfactant for seven consecutive days.

**IFO group:** Rats were orally given 1% tween 20 in DW for 7 days; on the seventh day, they received an intraperitoneal (IP) injection of IFO at a dose of 500 mg/kg.

**DZN + IFO group:** Rats received an oral DZN suspension (100 mg/kg/day) for seven consecutive days; on the seventh day, they were administered a single IP injection of 500 mg/kg IFO.

### Sample collection and preparation of brain tissue homogenate

On the eighth day, blood samples were collected from the external jugular vein, placed in a clot-activator gel tube, allowed to settle for about 15 minutes, and centrifuged at 3,000 rpm for 20 minutes [16]. The resulting supernatant (serum) was withdrawn by micropipette and transferred into Eppendorf tubes for the quantitative analysis of inflammatory markers (TNF-α, IL-6, iNOS) and the anti-inflammatory marker (IL-10) levels. The rats were anesthetized and euthanized through cervical dislocation. The brain was quickly extracted and washed in pH 7.4 PBS solution to remove blood residues. The brain tissue was then dried using filter paper, and the weight was measured before homogenization. For homogenization, the brain tissues were placed in a cold PBS solution (pH 7.4) at a ratio of 1:9 g/mL of tissue to PBS volume. This process was carried out using an electrical homogenizer. Subsequently, the homogenized samples were centrifuged at 10,000 rpm at a temperature of +4°C for 20 minutes. The collected supernatant was stored for subsequent quantitative measurement of Casp-3 enzyme levels [17].

### Statistical analysis

Data are reported as mean ± standard deviation (SD). The significance of differences among the various groups was assessed using one-way analysis of variance (ANOVA) conducted with SPSS software, version 25. A p-value of less than 0.05 was considered indicative of statistical significance.

## RESULTS

### TNF-α levels

**Figure 1:**
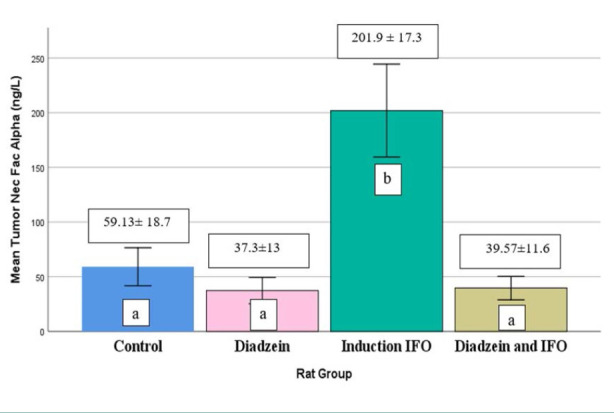
Serum TNF-α concentrations across treatment groups Data are expressed as mean±SD (n=7)

There was no significant difference in serum TNF-α levels between the DZN-only group and the control group, as illustrated in Table 1 and Figure 1. However, the IFO group had a significant increase in serum TNF-α levels compared to the control group. In addition, there was a decrease in serum TNF-α levels in the DZN + IFO group compared to the IFO group.

**Table 1:** Serum levels of inflammatory and anti-inflammatory markers

Group	No.	TNF-α (ng/L)	IL-6 (ng/ml)	iNOS (ng/L)	IL-10 (Pg/ml)
Control	7	59.13±18.7^a^	33.64±8.6^a^	3.77±0.44^a^	26.7±5.36^a^
DZN	7	37.3±13^a^	42±6.42^a^	3.7±0.47^a^	28.7±8.62^a^
IFO (500mg/kg)	7	201.9±17.3*^b^	91.2±16.56*^b^	6.67±1.16 ^*b^	63.2±8.9^*b^
IFO + DZN	7	39.57±11.6^a^	46.93±5.3^a^	4.4±1.04^a^	27.9±5.01^a^

Note: Values are mean±SD for each marker. Markers include TNF-**α**, IL-6, iNOS, IL-10. Statistical significance was assessed by one-way ANOVA.

(*) denotes a significant difference from the control group (p<0.05).

Different superscripts (a, b) within columns indicate significant differences between groups (p<0.05)

### IL-6 levels

**Figure 2:**
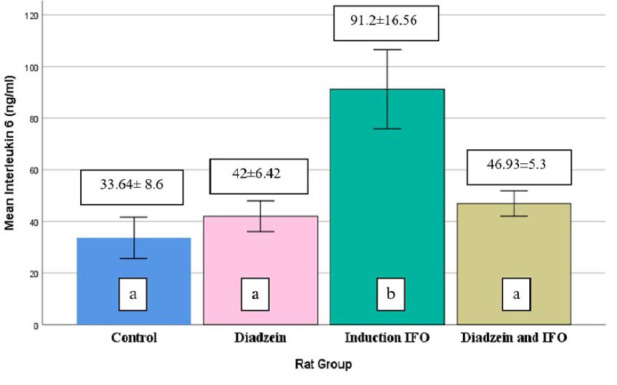
Serum IL-6 concentrations across treatment groups Data are expressed as mean±SD (n=7)

Serum IL-6 levels in the DZN group (100mg/kg/day) were not significantly different from the control group (Table 1 and Figure 2). However, there was a significant increase in serum IL-6 levels in the IFO group compared to the control. In addition, there was a decrease in serum IL-6 levels in the DZN+ IFO group compared to the IFO group.

### iNOS enzyme levels

**Figure 3:**
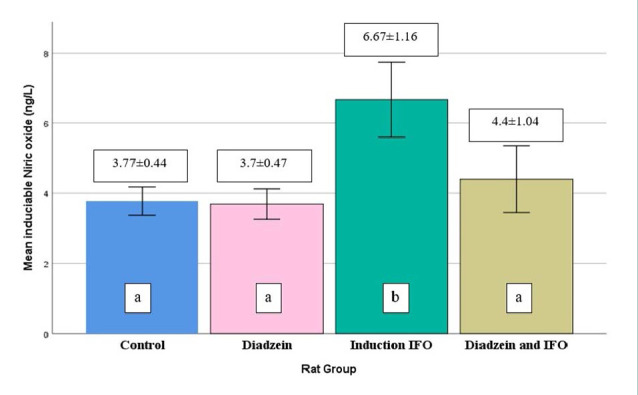
Serum iNOS concentrations across treatment groups Data are expressed as mean±SD (n=7)

No difference was observed in serum iNOS levels between the control and DZN groups (Table 1, Figure 3). The IFO group had significantly elevated iNOS levels compared to the control, while the DZN+IFO group had a significant reduction compared to the IFO group.

### IL-10 levels

**Figure 4:**
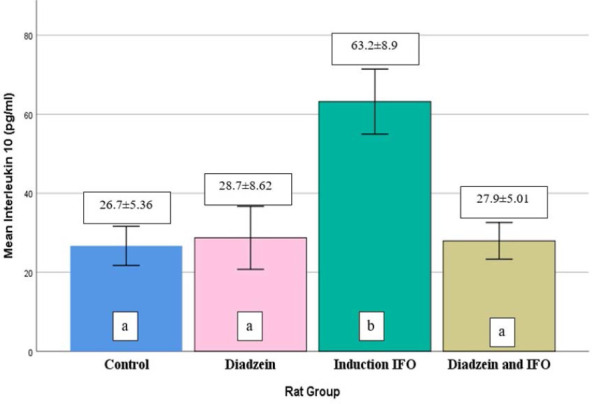
Serum IL-10 concentrations across treatment groups Data are expressed as mean±SD (n=7)

No difference was observed in serum IL-10 levels between the control and DZN groups, as demonstrated in Table 1 and Figure 4. The IFO group had significantly elevated IL-10 levels compared to the control. Additionally, the DZN+IFO group had a decrease in serum IL-10 levels compared to the IFO group.

### Caspase-3 levels

**Figure 5:**
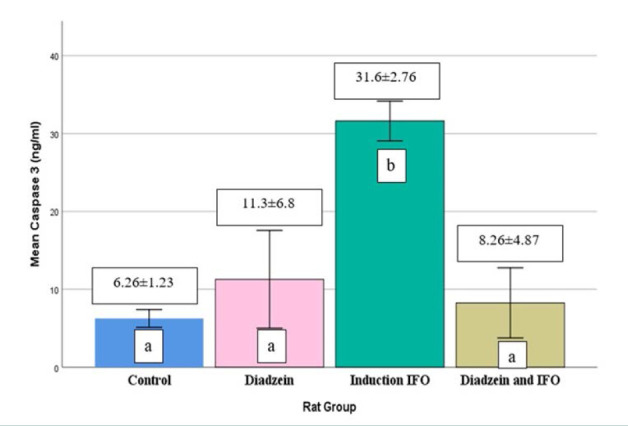
Caspase-3 levels in brain tissue across treatment groups Data are expressed as mean±SD (n=7)

There was no significant difference in caspase-3 levels in the brain tissue between the DZN and control groups (Table 2, Figure 5). However, the IFO group showed a significant increase in caspase-3 levels compared to the control. Conversely, the DZN+IFO group had a decrease in caspase-3 levels compared to the IFO group.

**Table 2:** Brain tissue levels of caspase-3 in each group

Group	No.	Caspase-3 (ng/ml)
Control	7	6.26±1.23^a^
Daidzein	7	11.3±6.8^a^
IFO (500mg/kg)	7	31.6±2.76^*b^
DZN + IFO	7	8.26±4.87^a^

Note: Values are mean±SD. Statistical analysis was performed using one-way ANOVA. (*) Indicates a significant difference from the control group (p<0.05). Different superscripts (a, b) within the same column denote significant differences between groups (p<0.05)

## DISCUSSION

IFO, an alkylating chemotherapy agent, is employed for treating various neoplasms, including sarcomas, lymphomas, and germ cell tumors [2]. While effective, IFO is known for adverse effects like alopecia, arrhythmias, interstitial pneumonitis, bone marrow suppression, hemorrhagic cystitis [3, 4], and neurotoxicity symptoms including fatigue, disorientation, and hallucinations [3, 5].

Neuroinflammation has been associated with neurological conditions and damage to the CNS induced by neurotoxicants [18]. Inflammatory mediators such as TNF-α and nuclear factor-κB (NF-κB) are crucial in the formation of neuroinflammation following chemotherapy [19]. Proinflammatory cytokines like IL-1 and IL-6, triggered by chemotherapy, can infiltrate the CNS from the peripheral immune system, causing localized brain inflammation [20]. Furthermore, nitric oxide (NO), which is involved in the pathophysiology of IFO-induced hemorrhagic cystitis in mice, is promoted by TNF-α and IL-1β, as reported by Ribeiro RA *et al*. [21]. NO, essential for CNS biochemical activities, is produced by different isoforms of nitric oxide synthase (NOS): neuronal (nNOS), endothelial (eNOS), and inducible (iNOS) [22, 23]. Typically, iNOS remains inactive in a healthy brain but becomes expressed in immune or glial cells in response to pathogens and increased cytokine production [24].

Our study demonstrated that IFO (500 mg/kg) significantly elevated serum TNF-α, IL-6, iNOS, and IL-10 levels compared to controls (Table 1, Figures 1-4). The increase in TNF-α and IL-6 corroborates the findings of Donegan *et al*. and Erta *et al*. [25, 26].

Another study showed that the anti-inflammatory properties of DZN were initiated through the suppression of the NF-κB signaling pathway with subsequent pro-inflammatory response suppression [27]. In the current study, IFO increased iNOS levels, supporting previous research that observed similar effects in rat liver [28]. This suggests that IFO may elevate iNOS enzyme levels across various tissues, not just in the brain. In addition, DZN oral administration for seven days prior to IP injection of IFO significantly reduced serum iNOS levels. These results differ from those of previous studies, making direct comparisons challenging.

Additionally, there is conflicting evidence regarding the activity levels of iNOS. Our findings align with the research conducted by Hämäläinen *et al*., where DZN was found to reduce the expression of iNOS in activated macrophages, attributed to the downregulation of STAT-1 LPS-induced and NF-κB activations [29]. Furthermore, the role of the 3′-hydroxyl group (3′-OH) in the chemical structure of DZN is crucial, as it contributes to scavenging nitric oxide (NO°) produced by iNOS action [30]. Conversely, another trial showed that DZN activated the iNOS and enhanced the production of NO° that resulted from the pathway of estrogen receptor (ER) in macrophages of RAW 264.7 [31].

In our study, we observed an increase in IL-10 in the IFO group, a finding not previously reported. In addition, IL-6 and TNF-α have been shown to initiate IL-10 synthesis by microglia in a dose-dependent pattern [32]. Moreover, DZN significantly decreased (p<0.05) the level of TNF-α and IL-6 in the IFO-induced neurotoxicity group (Table 1 and Figure 1 and 2, respectively).

Regarding the anti-inflammatory effect of DZN, in a separate study using mice as an animal model and 5-fluorouracil (5FU) to induce mucositis, the use of DZN resulted in the reduction of TNF-α levels, effectively mitigating inflammation [33]. Additionally, DZN has been shown to reduce pro-inflammatory cytokines and inflammation by inhibiting the excessive activation of astrocyte cells in rat models with focal ischemia of the cerebrum [27]. In this experiment, the oral use of DZN before IFO significantly reduced the serum level of IL-10 compared to such a serum level in the IFO group. These findings align with a study that employed a mice model and induced nephrotoxicity using cisplatin. In that study, DZN effectively reduced IL-10 levels, which was attributed to the modulation of dendritic cells by DZN [34-37]. There was an up-regulation of caspase-3 levels in the brain tissue of male rats IP injected with IFO, as shown in Table 2 and Figure 5. This finding suggests that apoptosis plays a significant role in the pathogenesis of brain damage induced by IFO. Our results align with similar findings reported in other studies [7, 8, 38].

An impaired balance between Bcl-2-associated X (Bax) and B-cell lymphoma 2 (Bcl-2) proteins results in the activation of the intrinsic (mitochondrial) apoptotic pathway. This imbalance leads to an increase in the levels of cytochrome-c, which is responsible for initiating the caspase cascade. The activation of caspase-3, primarily known for its role in the proteolytic degradation of a wide range of proteins, is a key step in this process, ultimately inducing apoptosis [36-38].

The administration of IFO led to the activation of the c-Jun N-terminal kinase (JNK) signaling pathway. This activation triggered the production of pro-inflammatory cytokines and initiated cell death through both intrinsic and extrinsic apoptotic pathways, as indicated by previous studies [7, 39]. Our current study found that oral administration of DZN one week prior to IFO exposure significantly reduced caspase-3 levels in the brain tissue of rats compared to those in the IFO-only group (p<0.05). Furthermore, the expression of Bcl-2 was increased by DZN, while the expression of the apoptotic regulator Bax (the apoptosis intrinsic pathway core regulator) was reduced. This led to a decrease in the release of cytochrome c and subsequently resulted in reduced caspase-3 levels, supporting other studies [40-43].

## CONCLUSION

In conclusion, DZN had anti-inflammatory and anti-apoptotic effects against the IFO-induced neurotoxicity in rats.
